# Self-affirmation and False Allegations: The Effects on Responses to Disclosures of Sexual Victimization

**DOI:** 10.1177/0886260520980387

**Published:** 2020-12-15

**Authors:** Melissa S. de Roos, Daniel N. Jones

**Affiliations:** 1 Roehampton University, London, UK; 2 University of Nevada, Reno, NV, USA

**Keywords:** sexual abuse, child abuse, reporting/disclosure, sexual assault, cultural contexts

## Abstract

The rise of the #MeToo movement highlights the prevalence of sexual victimization and gives a voice to victims who may have been silent before. Nevertheless, survivors or victims of sexual violence who come forward may be blamed or not believed. These reactions are evident both with adult and child victims. Further, fears about false accusations of sexual misconduct may negatively impact responses to disclosures. This study aimed to examine gender differences in perceptions toward the #MeToo movement, and the extent to which these translate into a skeptical response to disclosure. Further, we wanted to explore whether proximity to false allegations of sexual violence was linked with more negative responses and whether use of self-affirmations may decrease the likelihood of such a response. Through an online survey (*N* = 235) on Amazon’s Mechanical Turk, we assessed participants’ exposure to and perceptions of the #MeToo movement. Further, we asked them about their proximity to sexual violence (victimization or perpetration) and to false allegations. Using a threat manipulation (news article about false accusation) and a self-affirmation exercise, we studied the effects of both variables on responses to disclosure. Results indicated that after reading an article about a false accusation, male participants were more likely to blame a victim of childhood sexual abuse and to perceive the abuse as less harmful, compared with female participants. Further, we found that self-affirmation was linked with more supportive responses to a disclosure. These findings highlight the threatening nature of false accusations of sexual violence for men, and how this threat may shape the narrative regarding sexual violence. Opportunities to use self-affirmation to change this narrative to a more supportive one are discussed.

## #MeToo

In October 2017, the hashtag #MeToo became a worldwide trending topic on Facebook and Twitter (Strum, 2017). The phrase was originally coined by counselor Tarana Burke in 2006 to give a voice to victims and survivors^1^1The authors are aware of the ongoing debate about whether to use the term “victim” or “survivor” when referring to people who have experienced sexual violence (Schwark & Bohner, 2019). We believe that the decision should be the individual’s, but when discussing these people as a group, we have chosen to use “victim.” Using the term “survivor” assumes some form of recovery (Thompson, 2000), which the authors feel uncomfortable ascribing to all participants. of sexual violence, many of whom had not spoken out about their experience before. The purpose of reposting (sharing on one’s social media platform) the hashtag was to show the widespread prevalence of sexual violence and harassment, and to show other victims they are not alone. Victims of childhood sexual abuse (CSA) have also come forward about their experiences through using this hashtag (e.g., Alaggia & Wang, 2020). Within the first 24 hours, 4.7 million people worldwide interacted with the hashtag on Facebook (Santiago & Criss, 2017).

Despite the positive objectives behind the movement, perceptions of the movement vary. For example, recent research on perceptions of the movement found that men had more negative perceptions (Kunst et al., 2019), and this partially translated into a more skeptical response to a subsequent disclosure of sexual violence (de Roos & Jones, 2020). Further, some people believe the movement has gone too far and is ruining the lives of men who are publicly accused of sexual misconduct without due process (e.g., Stephens, 2017). Nodeland and Craig (2019) explored U.S. students’ perceptions of such accusations. They examined three high-profile accusations of sexual misconduct following #MeToo and found that women were more likely to support both legal and extralegal (i.e., termination of employment) repercussions than did men. Interestingly, having knowledge of the #MeToo movement predicted support of legal but not extralegal repercussions. This finding seems to contradict the characterization of the movement as a “witch hunt” (CBS News, 2018), but more research is needed on the subject.

Gender differences in responses to sexual violence are well-documented, with men more likely to respond with skepticism and victim blame (for a review, refer to Suarez & Gadalla, 2010). This gender difference may be due in part to proximity to sexual victimization (Cromer & Freyd, 2007; de Roos & Jones, 2020; Miller & Cromer, 2015; Nuttall & Jackson, 1994). Women are more likely to be close to someone who experienced sexual violence or to have experienced this themselves, leading to more supportive responses to disclosures of sexual violence (Miller & Cromer, 2015). Further, women may be more likely to disclose to other women. On the other hand, closeness to someone who perpetrated sexual violence may be linked with more negative responses to disclosures (de Roos & Jones, 2020).

More broadly, gender differences in response to sexual violence, and to victims of sexual violence, can be explained through social identity theory (Tajfel & Turner, 1979). According to social identity theory, when social categories such as age, gender, or ethnicity are threatened, for example, by being confronted with negative evaluation, members feel stronger ties to that category (e.g., Brown & Ross, 1982). This is particularly the case if that category is considered a minority group such as women, older people, or people of color (Grant, 1992). When under (perceived) threat, a group member may derogate the out-group to maintain their positive self-image (e.g., Judd & Park, 1988). Further, this negative reaction to the out-group may be directed at any member of that group, not just the member related to the immediate threat (Maass et al., 2003).

Focusing on gender, women have traditionally been regarded as a minority group, but initiatives such as #MeToo seek to empower women and to shift this imbalance (Mendes et al., 2018). Following from social identity theory, this change could lead men to identify more strongly with their gender and to respond negatively to disclosures of women’s sexual victimization.

## False Allegations

The perceived prevalence of false allegations of sexual violence may be particularly threatening to men. A commonly held view is that women and children lie about experiences of sexual victimization (Lonsway & Fitzgerald, 1994; Wheatcroft & Walklate, 2014). Lonsway and Fitzgerald (1994) argue that this idea is maintained by highly publicized cases of false allegations in the media. This perceived prevalence is not limited to the general population. Frontline law enforcement professionals, for example, tend to claim that false rape allegations are common (Brown et al., 2007; Gregory & Lees, 1996; Kelly et al., 2005; McMillan, 2018; Rumney, 2006; Spohne et al., 2014; Temkin, 2002). The combination of publicized cases and perceived prevalence may lend itself to certain cognitive biases. For example, the availability heuristic states that people’s perceived prevalence of a phenomenon is a result of how easily an incidence of the phenomenon can be recalled (Tversky & Kahneman, 1973). Thus, salience and prominence in one’s mind may lead to misperceptions of the frequency with which an event occurs and the severity of the consequences (Rothbart et al., 1978). As a result, the actual prevalence of false allegations of sexual violence may be less important for shaping an individual’s opinions than the subjectively perceived prevalence.

An additional hurdle to understanding the prevalence of false allegations is that documenting procedures for such cases are not uniform, resulting in mixed evidence (Lisak et al., 2010; Norton & Grant, 2008). Weiser (2017) points out that in the criminal justice system, a false allegation is classed as such if a claim is determined to be “unfounded.” However, this category includes baseless cases, which means a case does not meet the legal definition of sexual violence, or that the accuser may genuinely believe he or she was victimized but this later turns out not to be the case (Weiser, 2017). Thus, it appears that only a subset of false allegations as recorded in the criminal justice system represent cases where an intentional, false accusation is made.

Apart from official classifications, the decision to categorize an allegation as false may depend on other factors such as unwillingness to cooperate with an investigation, alcohol use on the part of the victim at the time of the assault (e.g., Archambault & Lonsway, 2012; Lisak et al., 2010), or other stereotypical beliefs about sexual violence (Lisak et al., 2010). As a result, an allegation categorized as “false” may actually be an honest allegation warranting investigation. Despite this ambiguity, experts agree that false allegations are not common or prevalent and that the more substantial problem is unreported cases of sexual violence (Fisher et al., 2000; Koss, 1988; National Victim Center, 1992; Saunders, 2012). Similarly, with regards to false allegations of CSA, barring specific circumstances such as child custody hearings, most allegations of CSA are founded (O’Donohue et al., 2018).

Finally, when considering false allegations as a threat, it is important to point out that most cases of false allegations involve an allegation of stranger rape, presumably because such cases are consistent with people’s stereotypical expectations of sexual violence, and thus may be perceived as more credible (Kelly, 2010). O’Neal et al. (2014) found that a perpetrator was named by a victim in about 18% of false allegations, and arrests in these cases represented about 2.8%, with .9% leading to a conviction. False allegations do occur, but nevertheless, biases, such as the availability heuristic may lead to the perception that they are more widespread than is warranted. Regardless of any factual basis, the perceived prevalence of false allegations is likely to play a role in an individual’s response to a disclosure, and men, in particular, may be more sensitive to this issue as it could be perceived as a threat.

## Self-affirmation

A defensive response impedes the ability to change attitudes toward victims and sexual violence. However, several studies have explored how threats to the self can be alleviated to facilitate positive change. Self-affirmation theory’s basic tenet is that people strive to maintain a consistent view of themselves as moral and just (Cohen & Sherman, 2014; Steele, 1988). Self-affirmations then, are tactics used to maintain this view. An example of such a tactic is to let the individual think about a personal characteristic they value in themselves and to write about a time when this value was important (Harber, 1995). Self-affirmation has been shown to facilitate participants’ willingness to attend to information that may be threatening to the self (Cohen et al., 2000; Nelson et al., 2014; Schumann, 2014) and thus may be a useful tactic to prevent a defensive response to a perceived threat.

Self-affirmation strategies have also been employed in social psychological research. Following self-affirmation, people were more likely to help other people in need (Kim & McGill, 2018). Further, awareness of other people’s pain may be ignored to avoid feeling uncomfortable (Batson et al., 1997), but by affirming the self, such avoidance dissipates. A series of studies demonstrated that self-affirmation increased participants’ willingness to help others and it made participants more likely to pay attention to information about other people’s struggles (Kim & McGill, 2018).

Self-affirmation may be especially helpful in situations where people’s identity or group identity is threatened. For example, Phillips and Lowery (2015) found that when White participants were exposed to information that highlights White privilege, these participants exaggerated the personal struggles they had endured to a greater extent than White participants who had not read this information. Interestingly, this effect was reversed if participants engaged in self-affirmation before reading the information. This finding is especially relevant to the current research because #MeToo can be interpreted in a similar manner. Specifically, the highlighting of issues that disproportionately affect women may cause men to defensively emphasize their own struggles, perhaps in the form of the risk of being falsely accused of sexual violence. It would be expected then that self-affirmation may prevent this defensive process from being triggered.

The most common self-affirmation manipulation is writing about personal values. In doing so, participants remind themselves of being moral and just and are less likely to feel threats to their integrity (e.g., Master et al., 2009). Participants are also less defensive following self-affirmation (refer to Sherman & Cohen, 2006, for a review). Thus, a balanced message that incorporates self-affirmation while continuing to raise awareness about the prevalence of sexual violence will be more likely to yield the desired change in attitudes about sexual violence, and thus facilitate a safe environment for victims and survivors to come forward.

## CSA Disclosures

One of the criticisms of #MeToo is that it excludes victims who deviate from expectations about victims (Kessler et al., 2019). For example, people of color (Onwuachi-Willig, 2018), people who work in the sex industry (Farley, 2018), or non-heterosexual people (Ison, 2019) seem excluded from the narrative. The same observation can be made about child victims (Agathis et al., 2018). CSA remains a prevalent, worldwide problem (Pereda et al., 2009). Despite this prevalence, child victims are not immune to skeptical responses to disclosures. High-profile cases over the last decade indicate that children who disclose sexual victimization to an adult are sometimes not believed, and as a result, the abuse continues (Arata, 1998). Given that disclosure during childhood is rare, the importance of believing children cannot be overstated.

## The Present Study

The first goal of this study was to replicate some of the findings of an earlier study regarding exposure to and perceptions of the #MeToo movement (de Roos & Jones, 2020), and to further expand on these findings by adding false allegation and self-affirmation as variables. Our second goal was to determine whether a threat of false allegation would affect how people respond to a disclosure. Due to the importance of a perceived threat on triggering defensive attributions, we included a threat condition describing a case of a false accusation of sexual violence and the consequences to the accused. To our knowledge, no other study has examined how perceptions of false allegations affect people’s responses to disclosures of sexual victimization. We expected that male participants would feel threatened by a false allegation and respond more negatively to a subsequent disclosure. We assessed proximity to sexual violence as well as perceived proximity to false allegations to examine whether an availability heuristic relating to false accusations may underlie responses to a CSA disclosure. We expected that proximity to victims of sexual violence would result in more supportive responses to a disclosure, whereas proximity to perpetrators or to perceived false allegations should result in more skeptical responses. These effects should remain even when controlling for participant empathy. Finally, our third goal was to examine whether a self-affirmation exercise protects against defensive responses, resulting in a more supportive response to a disclosure.

## Methods

### Participants

Participants were recruited through Amazon’s Mechanical Turk. MTurk has been used to conduct research with a non-student population. The data collected through MTurk tend to be as reliable as data collected in student populations (Buhrmester et al., 2016). Restrictions were set to include only participants located in the United States, who had an acceptance rate of their surveys of 95% or higher, and who had at least 50 accepted participations. A power analysis indicated that 200 participants were required to detect the desired effect size (*R*^2^ = .12), taken from a previous study (de Roos & Jones, 2020). A total of 250 participants who followed instructions for the self-affirmation or control task and who correctly answered the fact-based questions regarding the article they read were included. Participants were compensated $1.00 for their participation. Participants took an average of 14 minutes to complete the survey.

The mean age in this sample was 37.74 years (*SD* = 12.92). Male participants represented 47.2% of this sample, and female participants 52.0%. Two participants identified as non-binary (.8%). Unfortunately, this number is too low to include in analyses and as such, these participants were excluded from analyses addressing gender differences. The majority of our participants identified as straight (88.8%), with 8.8% identifying as bisexual, and 1.6% as gay or lesbian. Most participants had a college degree (79.2%), with 15.6% having attended college but not obtained a degree. The remainder of participants had a high school degree or equivalent (5.2%). A majority of participants were employed either full-time (66.8%) or part-time (21.2%). The rest of the participants were either unemployed (8.8%) or retired (3.2%). Most participants identified as White (79.2%; 12.4% of total sample identified as Hispanic), Black (10.8%), or Asian (7.2%). The remaining participants identified as Mixed Race (2.0%) or American Indian or Alaskan Native (.8%). Finally, 58% of participants indicated they had children.

### Measures

#### CSA vignettes.

Vignettes depicting realistic scenarios are considered the preferred way to assess responses to sexual violence compared with self-report questionnaires (Font, 2013). The same vignette from an earlier study by de Roos & Jones, 2020 was used and can be found in supplemental materials. After reading the vignette, participants were asked the extent to which they believed the CSA disclosure (rate on scale of 1–100), how harmful they thought this experience was (1 = *Not at all harmful*, 5 = *Very harmful*), and how much Zoe, Zoe’s mom, and Zoe’s mom’s boyfriend were to blame (rate on scale of 0–100).

#### False allegations threat manipulation.

The threat manipulation was a brief news story adapted from a real BBC news story (BBC News, 2010) detailing a man whose life was ruined after his ex-girlfriend falsely accused him of rape. The article can be found in supplemental materials. The article mentions an increase in reporting of sexual assault following #MeToo and that 1 of 428 reported cases turned out to be false, but all the other accused parties were found guilty. Although these numbers do not reflect a realistic scenario, such an extreme figure was chosen to examine whether this one false allegation has a greater effect than the 427 allegations that were proven. The control condition consisted of a news article reporting on #MeToo as an important campaign raising awareness (Nicora, 2017). It highlights the problem of underreporting, and some of the challenges survivors face when considering coming forward with their experiences. This article has also been included in supplemental materials.

#### Self-affirmation task.

The self-affirmation exercise used in this study was adapted from Harber (1995) and Cohen et al. (2000). It presents participants with a list of characteristics and values and asks them to pick the one they feel is most important to them. They are then asked to remember an occasion when that value or characteristic was important to them and made them feel good about themselves. The control condition was the same as the one used by Cohen et al. (2000). It asked participants to list everything they had to eat or drink in the past 48 hours. This manipulation is a preferred control condition to asking participants to write about a value not important to them, as those prompts still tend to evoke self-affirming reflections.

#### PANAS-short form.

The 10-item PANAS-SF (Thompson, 2007) was used to assess participants’ emotional responses to the article. It presents participants with positive (i.e., Active) and negative (i.e., Upset) emotions and asks them to indicate the extent to which they feel the emotion (1 = *Very slightly or not at all*, 5 = *Extremely*). Internal reliability in this sample was good (*α* = .84).

#### Toronto Empathy Questionnaire.

The Toronto Empathy Questionnaire (TEQ: Spreng et al., 2009) was used to assess empathy. The TEQ consists of 16 items (i.e., “I get a strong urge to help when I see someone who is upset”) that are scored on how often the participant experiences the item (0 = *Never*, 4 = *Always*). Items are summed to create a total empathy score. Internal reliability in this sample was good (*α* = .87).

#### #MeToo exposure.

The same questionnaire from an earlier study was used (de Roos & Jones, 2020). Before asking participants about #MeToo, they were asked if they knew anyone who was a victim of sexual violence (yes/no/would rather not say). The Inclusion of Other in the Self Scale (IOSS: Aron et al., 1992) was then used to assess the participant’s closeness to this person. This scale presents participants with seven images of two circles that increase in overlap. The circles represent the participant and the other person. Participants are then asked to pick the set of circles that best describes their relationship with the other person. This scale correlates highly with other measures of perceived closeness of relationship and assesses this construct reliably in MTurk populations (Gächter et al., 2015).

Following this, participants were asked if they had heard of the movement and if they had reposted (shared on one’s social media platform) the status themselves. Participants were then asked if they knew someone who had reposted the status, someone who did something that would make someone else repost the status, someone who was falsely accused of committing sexual violence, or someone who falsely accused someone of sexual violence. For each of these questions, if participants knew such a person, they used the IOSS to indicate their closeness to these people. Finally, if participants reposted the status themselves, and indicated that they knew someone who reposted the status, we asked them if the person they knew was the same person who did something to make *them* repost the status.

#### # MeToo perceptions

To assess perceptions of the #MeToo movement the same 12-item questionnaire as in an earlier study (de Roos & Jones, 2020) was used. It asks participants about their perceptions of, and feelings about #MeToo. The items are based on criticisms of the movement (i.e., Witch Hunt) as well as on its goals (Empowering). For each item, participants indicate the extent to which they agree with each statement on a 5-point Likert scale (1 = *Strongly Disagree*, 5 = *Strongly Agree*). Internal reliability for both positive (*α* = .91) and negative perceptions (*α* = .89) was good. The full questionnaire is included in supplemental materials.

### Procedure

Participation occurred through Amazon’s Mechanical Turk. Following agreement to consent, participants were randomly assigned to complete the self-affirmation or control task. They were then randomly assigned to read the threat manipulation article or the control article. Participants then answered two questions to ensure they paid attention to the article, and they filled out the PANAS-SF. On the next screen, participants were presented with the vignette. They were asked to carefully read the vignette before moving onto the next screen. Participants were then asked the extent to which they believed the vignette, how harmful they thought the experience was, and who was to blame. On the next screens, participants were asked to complete the #MeToo questionnaire and the TEQ. Finally, participants were debriefed and thanked for their participation.

## Results

### Replication

More than half of participants reported knowing a victim of sexual violence (54.0%), independent of #MeToo. Women (66.9%) were significantly more likely to know a victim of sexual violence than men (39.8%) (*X^2^* (1, *N* = 232) = 19.37, *p* < .001), and to be closer to a victim than men (*M*_male_ = 4.19, *SD*_male_ = 1.90; *M*_female_ = 5.10, *SD*_female_ = 1.76; *t* = –2.79, *p* < .001).

The majority of participants had heard of the #MeToo movement (86.0%), and 21.6% reposted the status themselves. Two-thirds of participants knew someone who reposted the status and 22% reported knowing someone who did something that would make someone else repost. Of the people who reposted #MeToo, 26 (83.9%) answered the closeness to a perpetrator question about the person who did something to make them repost the status, and their mean closeness to the perpetrator was 4.72 (*SD* = 1.65). No significant gender differences were found in exposure. Participants who had not heard of the movement were excluded from analyses with #MeToo variables.

An independent samples *t*-test was conducted to examine whether there were differences in perception of the movement between men and women. Male participants were significantly more likely to have negative perceptions of #MeToo (*M* = 17.52, *SD* = 7.05) than were women (*M* = 13.92, *SD* = 5.64; *t* = –3.29, *p* = .001). Similarly, women (*M* = 24.32, *SD* = 5.47) were significantly more likely to have positive perceptions of #MeToo than men (*M* = 22.04, *SD* = 4.52; *t* = 4.07, *p*<.001).

Correlational analyses were conducted to examine the relation between perceptions of #MeToo and responses to vignette. Results are displayed in Table 1. Participants with positive perceptions were more likely to believe the vignette and to view it as harmful, and more likely to blame the perpetrator. Participants with negative perceptions responded more negatively across all variables.

**Table 1.** Correlations Between Perceptions of #MeToo and Responses to a CSA Vignette.

	Positive Perceptions	Negative Perceptions
Believe	.49***	–.29***
Blame Zoe	–.02	.58***
Blame BF	.23**	–.41**
Blame mom	–.03	.35***
Harmful	.33**	–.36***

*Note*. **p< .01, ***p< .001, ^†^*p*< .10.

Finally, the correlations between exposure to #MeToo and perceptions of #MeToo were examined. Having reposted the status was associated with stronger positive (*r* = .17, *p* = .01) and negative (*r* = .40, *p* < .001) perceptions. Being close to someone who reposted the status was linked to positive perceptions (*r* = .28, *p* < .001), whereas being close to someone who did something to make someone else repost had the opposite effect (*r* = .39, *p* < .001). Similar results were found for proximity to sexual victimization independent of #MeToo, such that being close to a victim of sexual violence (*r* = .27, *p* < .001) was related to positive perceptions of #MeToo.

### False Allegations

With regards to false accusations of sexual violence, 16% of participants reported knowing someone who was falsely accused and 14.4% of participants knew someone who falsely accused someone. Men (25.4%) were more likely to report knowing someone who was falsely accused of sexual violence than women (13.1%; *X^2^* (1, *N* = 248) = 6.14, *p* = .01). Similarly, men (24.6%) reported knowing someone who had falsely accused someone else more than women did (6.9%; *X^2^* (1, *N* = 235) = 14.47, *p* < .001). Knowing someone who was falsely accused (*r* = .57, *p* < .001) or who falsely accused someone (*r* = .43, *p* < .001) was linked to more negative perceptions of #MeToo.

We hypothesized that male participants would feel threatened by the false allegation article and thus respond more negatively to a subsequent CSA disclosure. Composite scores were created from PANAS items to create two separate indices of negative and positive affect. We then conducted two ANOVA’s with gender and article condition as predictor variables. For positive affect, a significant main effect was found for gender (*F*(1, 238) = 11.68, *p* = .001) such that female participants (*M* = 24.09, *SD* = 5.61) experienced more positive affect than male participants (*M* = 21.96, *SD* = 4.48). Similarly, a significant main effect was found for article condition (*F*(1, 238) = 4.63, *p* = .03) such that participants who read the control article (*M* = 23.84, *SD* = 4.48) experienced more positive affect than those who read the false allegation article (*M* = 22.35, *SD* = 5.81). For negative affect, a significant interaction effect was found (*F*(1, 236) = 6.51, *p* = .01). Male participants who read the threat article (*M* = 19.31, *SD* = 6.42) reported significantly more negative affect than female participants who read the threat article (*M* = 14.05, *SD* = 4.97).

**Figure 1a.**
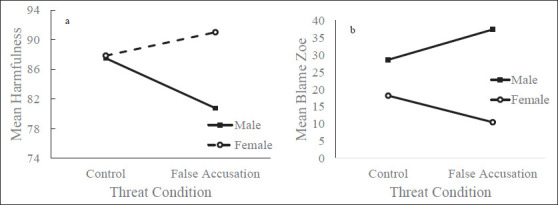
The effect of article condition on the amount of blame allocated to Zoe for male and female participants., Figure 1b. The effect of article condition on the perceived harmfulness of the vignette for male and female participants.

*Note.* Women represented by dashed line in both figures.

A series of two-way ANOVA’s with gender and article condition were conducted to examine if there were any interaction effects on belief in the vignette, blame allocation, and perceived harmfulness. A significant interaction effect was found for judging the vignette as harmful (*F*(1, 239) = 6.30, *p* = .01; Figure 1a). A similar interaction was found for the amount of blame attributed to the victim (*F*(1, 240) = 4.47, *p* = .04; Figure 1b). Both of these interactions remained significant after entering closeness to a victim of sexual violence as a covariate (Harmful: *F*(1, 238) = 7.81, *p* = .006; Blame Zoe: *F*(1, 238) = 5.93, *p* = .02).

### Proximity to Sexual Violence and False Allegations

To assess whether proximity to sexual violence or perceived false allegations affected how people responded to a CSA disclosure, a series of multiple regressions were conducted. For each, closeness to someone who reposted the status, someone who did something that would make someone else repost the status, someone who was falsely accused of sexual violence, someone who falsely accused someone of sexual violence and empathy were added. We also added closeness to a victim of sexual violence independent of #MeToo to determine whether this variable operated differently from closeness to someone who reposted the status. Finally, we included participant gender.

A significant model was found for believing the vignette (*R*^2^ = .20) but empathy (*β* = .37, *p* < .001) was the only significant predictor. With regards to the amount of blame allocated to Zoe, a significant model was found (*R*^2^ = .51). First, both closeness to someone who reposted the status (*β* = .21, *p* < .001), and closeness to a victim independent of #MeToo (*β* = –.24, *p* < .001) were significant predictors. Second, closeness both to someone who was falsely accused (*β* = .26, *p* < .001) and someone who falsely accused someone else (*β* = .17, *p* = .02) were significant predictors. Finally, empathy entered the model as a significant predictor of belief in the vignette (*β* = –.32, *p* < .001). For blaming Zoe’s mom’s boyfriend (*R*^2^ = .36), closeness to someone who reposted the status (*β* = –.18, *p* = .01), closeness to a victim of sexual violence independent of #MeToo (*β* = .21, *p* = .001) and empathy (*β* = .45, *p* < .001) were significant predictors. The only significant predictor that emerged in the model predicting amount of blame allocated to Zoe’s mom (*R*^2^ = .12) was closeness to someone who did something that would make someone else repost the status (*β* = .18, *p* = .03). Finally, a significant model was found for the perceived harmfulness of the vignette (*R*^2^ = .26), but empathy (*β* = .45, *p* < .001) was the only significant predictor. Excluding participants who answered the question about closeness to someone who did something that would make someone repost about the person who made *them* repost did not change these results.

### Self-affirmation

To determine whether self-affirmation affected participants’ responses to a CSA disclosure, a series of ANOVA’s with self-affirmation and false accusation as predictors were conducted. No interaction effects emerged, however, a significant main effect of self-affirmation was found on the amount of blame allocated to Zoe (*F*(1, 242) = 4.79, *p* = .03), such that participants in the self-affirmation condition (*M* = 18.81, *SD* = 29.28) were less likely to blame Zoe than those in the control condition (*M* = 28.16, *SD* = 33.86). An effect was found for the amount of blame allocated to Zoe’s mom’s boyfriend (*F*(1, 242) = 10.29, *p* = .002), such that participants in the self-affirmation condition (*M* = 93.03, *SD* = 13.92) were more likely to blame Zoe’s mom’s boyfriend than those in the control condition (*M* = 86.08, *SD* = 18.25). Finally, a significant main effect was found for perceived harmfulness of the vignette (*F*(1, 241) = 7.28, *p* = .007), such that participants in the self-affirmation condition (*M* = 89.71, *SD* = 14.13) perceived the vignette as more harmful than those in the control condition (*M* = 84.39, *SD* = 16.44).

## Discussion

The goal of this study was to replicate some of the findings from an earlier study (de Roos & Jones, 2020) and to extend them to include the threat of false allegations, and the possible effect of self-affirmation on defensiveness. Consistent with previous research, we found that male participants were more likely to hold negative perceptions of the movement than female participants. Further, these perceptions were related to responses to a CSA disclosure, such that participants with more negative perceptions were less likely to respond in a supportive manner. Replicating previous research, we found that reposting the #MeToo status was associated with overall stronger feelings, both positive and negative. This is consistent with previous research. Mendes et al. (2018) interviewed people who used a similar hashtag to publicly disclose sexual victimization (#RapedButNeverReported) and found similar mixed emotions. On the one hand, participants reported receiving supportive responses, but on the other hand, they were also met with hostility and online abuse. Future research could explore the motivations for people reposting the status, as well as how the responses they received affected them. Finally, participants who reposted acknowledge themselves as a victim, which may indicate greater well-being or adjustment. Future studies should include questions about why participants may have chosen not to repost the status.

With regards to proximity to a victim or to someone who did something that would make someone else repost, our findings were consistent with previous research. Closeness to a victim was associated with positive perceptions, and closeness to someone who did something that would make someone else repost was linked with negative perceptions, again, underlining the importance of proximity for shaping perceptions (de Roos & Jones, 2020).

We asked participants about their subjective experience with false allegations of sexual violence. Results indicated men were more likely to report knowing someone who was falsely accused of sexual violence. This finding is consistent with expectations about who is more likely to be accused of sexual violence and is a potential trigger for defensiveness in men concerning #MeToo. We did not ask any further questions about the nature of such a false allegation, thus relying on the participant’s judgment of whether an allegation was indeed false. It may be the case that “false allegation” was confounded with “unfounded” allegation. Further, we emphasized the participant’s perception of whether the allegation was false, rather than whether an allegation was actually false, and thus our conclusion pertains to people’s subjective experience with false allegations of sexual violence. A future study may ask participants to discuss the nature of their experience with a false allegation to differentiate between intentional false accusations and unfounded accusations, as well as explore their perceptions about the experience in more depth.

The addition of proximity to false accusations showed that this factor, whether it was knowing an accuser or accused, was related to more negative perceptions. Thus, subjective knowledge of a case of false allegation may negatively skew people’s perceptions of the #MeToo movement. It would appear then that people who have a negative experience with false accusations generalize this experience to a movement, and display more skeptical views as a result. With the current media narrative surrounding false allegations, this is particularly important, as it is likely that this contributes to an overestimation of the prevalence of false allegations, consistent with the availability heuristic. As a result, skepticism toward victims may increase. Future research may ask participants about some commonly held myths surrounding false allegations, such as high prevalence rates, to ascertain how such ideas about false allegations in general affect responses to a specific disclosure.

The article mentioning a false accusation of sexual violence was included to create a threat that may enhance defensive attributions, particularly in male participants. Consistent with social identity theory, this manipulation elicited the expected emotions, with men reporting significantly more negative emotions than women after reading the false accusation article. This finding supports the idea that men perceive the potential for false allegations as a threat, resulting in negative emotions. The expectation was that as a result of such emotions, male participants would be more likely to respond negatively to a CSA vignette after they read the false accusation article. Indeed, results indicated that men who read the false accusation article were more likely to blame Zoe for what happened and to perceive the vignette as less harmful. These particular variables were also impacted in other research (de Roos & Jones, 2020), suggesting that defensiveness may be a secondary process that stems from a threat to one’s social identity (in this case, one’s gender identity). Belief in the vignette is not affected, but participants then cope with these mixed feelings of belief and defensiveness by minimizing harm and blaming the victim. Future research might address whether this is a functional strategy. For example, Maass et al. (2003) assessed how male participants responded to an identity threat to their gender, made by a woman. When participants had the option to “punish” this woman, they reported greater identification with their gender than if they had not taken this option. Similarly, blaming the victim in this study may have made male participants who read the threat article feel better. More broadly, it may be the case that because false allegations are viewed as a threat, blaming the victim and minimizing the harmfulness of sexual violence serve to protect men’s gender identity. The article did misrepresent the reality of the number of cases that are proven in court, which could have skewed participants’ views. A future study may wish to include a more realistic scenario to assess the effect of threat of false allegations.

Similarly, both beliefs in the vignette and its perceived harmfulness were not influenced by proximity to sexual violence or false allegations. Instead, empathy was the only predictor, suggesting again that believing a disclosure and judging harmfulness are different processes from allocating blame for the incident. The findings for blame allocated to victim and perpetrator showed a different pattern. Participants who were close to a false accuser or a person falsely accused of sexual violence were more likely to blame the victim. With regards to the amount of blame allocated to the perpetrator, proximity to false allegations did not play a significant role. This finding is interesting for two reasons. First, it shows that blame allocated to victim and perpetrator are not complimentary; blaming the victim more, does not automatically involve blaming the perpetrator less. Second, the victim derogation in the absence of absolving the perpetrator suggests that individual victims may become the target of defensive responses to disclosures of sexual victimization.

Interestingly, closeness to someone who reposted #MeToo was linked with more blame allocated to the victim, but the opposite effect was found for closeness to a victim of sexual violence independent of #MeToo. This indicates that the movement may indeed be polarizing, with the mere association of #MeToo leading to more victim blame than actually knowing a victim. However, this is consistent with previous research that highlights polarization of views when the privileged group feels that their privilege is challenged (Blais & Dupuis-Déri, 2012). Clearly, while #MeToo may highlight the prevalence of sexual victimization, it does not enhance victim empathy to the extent that it makes people more supportive of victims. Similarly, closeness to someone who reposted #MeToo was linked with blaming the perpetrator less, but again, the opposite effect was found for knowing a victim of sexual violence independent of #MeToo.

Finally, self-affirmation was hypothesized to buffer the triggered defensive attributions in male participants. Despite the absence of a main effect for article condition, the effects of self-affirmation, the article, and the interaction between the two on the vignette variables were assessed. No interaction between article and self-affirmation emerged, but self-affirmation did have a positive effect on the three variables that this study’s results suggested may be part of a defensive response; blame allocated to the victim, to the perpetrator, and the perceived harmfulness of the vignette. In each of these cases, participants in the self-affirmation condition were more likely to respond in a supportive manner. These findings are in line with previous research that found that self-affirmation can reverse a defensive response to information that threatens the individual’s social identity (Sherman & Cohen, 2002). As such, self-affirmation may be a useful tool in countering defensive responses to sexual violence and disclosures.

The results on self-affirmation have implications for the success of a public awareness campaign, such as #MeToo. The results underline the importance of empowering male allies and making them feel as if they can be part of the solution rather than the problem (Banyard et al., 2004). In-group members dictate what is considered (un)acceptable behavior to the group (Pettigrew, 1961; Turner, 1991). As such, men calling out men for inappropriate behavior is the best way to reshape social norms surrounding acceptable behavior, thus transforming from defensiveness to supportiveness (Fabiano et al., 2003). Maass et al. (2003) highlighted the importance of changing what it means to be a man, and change the perceived threat to identity to something consistent with that identity. Indeed, several interventions have used this strategy to include being supportive of victims of sexual violence, whilst exclude derogatory behavior from male identity norms (Gotell & Dutton, 2016; Harlow et al., 2018).

Attempts at engaging men and boys are evident in hashtags such as #HowIWillChange, which trended in Twitter in response to #MeToo (Vagianos, 2017). As a recent study points out (PettyJohn et al., 2018), hashtags usually focus on giving a voice to women who experience violence at the hand of men, for example, #MaybeHeDoesn’tHitYou, #WhyIStayed, #WhyILeft, #YesAllWomen (Cravens et al., 2015; Maas et al., 2018; McCauley et al., 2018; Rodino-Colocino, 2014). These hashtags leave no room for men to join the conversation and to evaluate their own role in the perpetuation of violence against women and girls. #HowIWillChange was started by a man, with the intent of engaging boys and men in the conversation. PettyJohn et al. (2018) conducted a qualitative analysis of the hashtag to examine the content and how people were talking about it. They identified three themes; actively engaging to attempt to dismantle rape culture, resistance to social change because it is perceived as unfair to men, hostile resistance to social change. It would appear that only the first theme is indicative of a response free from defensiveness. Perhaps incorporating self-affirmation in the conversation will allow men to sidestep defensive responses and respond in this more supportive manner instead. Future studies may use some of these tweets to examine how it makes people respond, and what type of discourse is more useful in engaging men as allies.

### Limitations and Future Directions

This study had some limitations. We did not ask about participants’ own perpetration. Further, the lack of details about the information recalled regarding victimization, perpetration, and false allegations means that a wide range of circumstances may have been included in participants’ answers. Future studies should incorporate questions that ask about more specific circumstances to explore the types of experiences participants identify as relevant. Although Amazon’s MTurk could provide a more diverse sample than an undergraduate student sample, MTurk samples are not necessarily representative of the U.S. population (Arditte et al, 2016). The threat manipulation may have also been too extreme and the self-affirmation manipulation not strong enough. Future research may wish to use a false accusation vignette where the life of the accused was not ruined. Similarly, providing a stronger self-affirmation manipulation such as by giving feedback to participants on their compassion, may have increased the impact of self-affirmation. Finally, we did not ask participants’ responses to the disclosure before or after the manipulation threat, and thus we cannot conclude that exposure to a threatening article caused some participants to respond more skeptically to the disclosure.

This study replicated and extended the findings of our first study and found similar gender differences in exposure to sexual victimization and perpetration, as well as in perceptions of CSA disclosure and the #MeToo movement. People who knew someone who had experienced false accusations were more likely to hold negative views of the #MeToo movement. Threatening men with a story about a false allegation appeared to have some effect on more victim-blaming as well as minimizing the harmfulness of the CSA scenario. Regardless of threat, self-affirmation reversed the defensive process in making participants respond in a more supportive manner. Findings highlight the impact of exposure to false allegations, whether or not these allegations are truly false or confounded, and how this could lead to skepticism in response to a disclosure of sexual violence. Using self-affirmation may be a promising strategy to engage men in the conversation about sexual violence, reducing the likelihood of a defensive response.

## Supplemental Material

Supplemental material for this article available online. Click here for additional data file. Supplemental Material for Self-affirmation and False Allegations: The Effects on Responses to Disclosures of Sexual Victimization by Melissa S. de Roos, Daniel N. Jones, in Journal of Interpersonal Violence
