# Supplementary material for: Self-affirmation and False Allegations: The Effects on Responses to Disclosures of Sexual Victimization
Source: J Interpers Violence. 2020 Dec 15;37(11-12):NP9016–39. doi: 10.1177/0886260520980387 (PMC9136472; doi:10.1177/0886260520980387)
Supplement: Supplementary file 1 — Supplemental material for this article available online. [file sj-pdf-1-jiv-10.1177_0886260520980387.pdf]

#metoo Exposure & Perceptions

1. Do you know anyone who is a victim/survivor of sexual violence?
    a. Yes
    b. No
    c. Prefer not to say
2. Think of the person you know who is a victim/survivor of sexual violence that you feel closest to. Which of the below images best represents your relationship with this person?

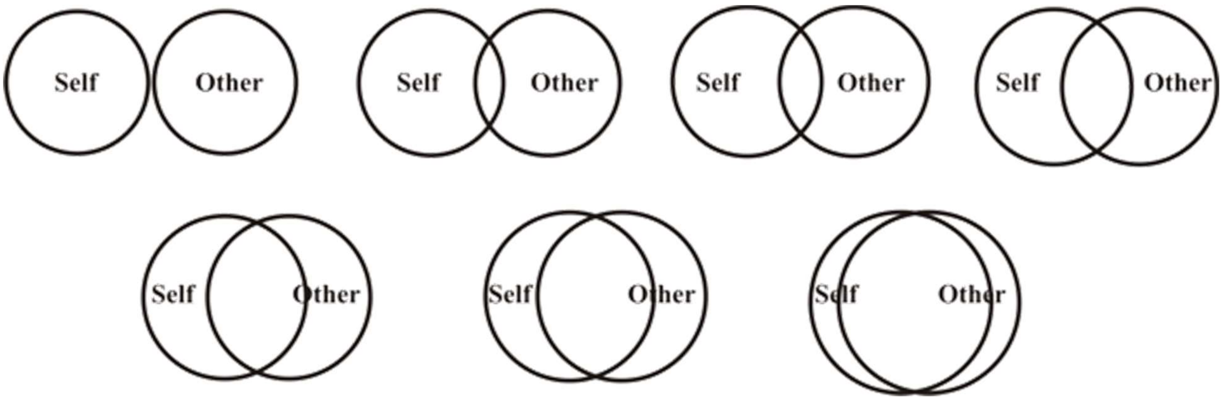

3. Have you heard of the #metoo movement?
    a. Yes
    b. No
4. Have you ever reposted #metoo online?
    a. Yes
    b. No
    c. Not sure/I'd rather not say
5. Do you know someone else who reposted #metoo?
    a. Yes
    b. No
    c. Prefer not to say
6. Think of the person you know reposted #metoo that you feel closest to. Which of the below images best represents your relationship with this person?

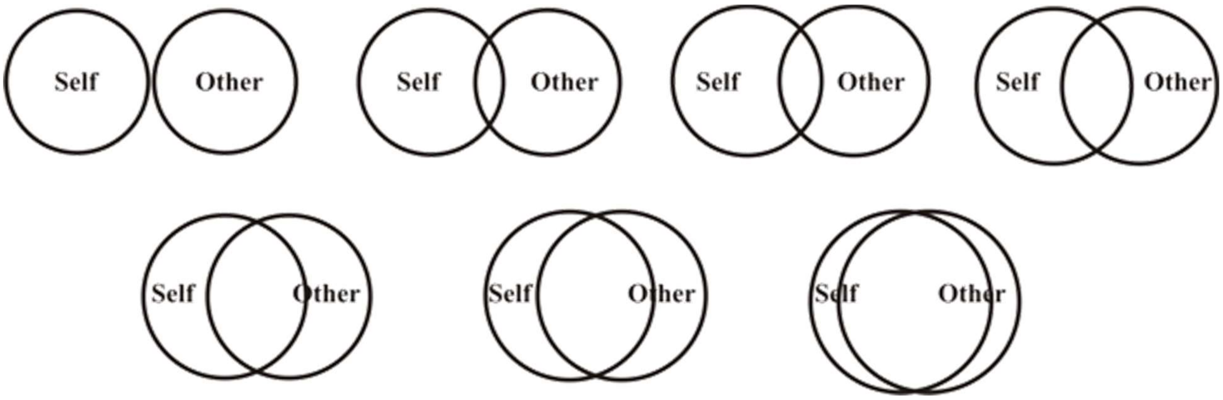

7. Do you know someone who has engaged in anything that might cause someone else to repost #metoo? (i.e. sexual harassment, sexual violence, sexual assault, rape)
    a. Yes
    b. No
    c. Not sure/I’d rather not say

8. Think of the person you thought of in the previous question. Which of the below images best represents your relationship with this person?

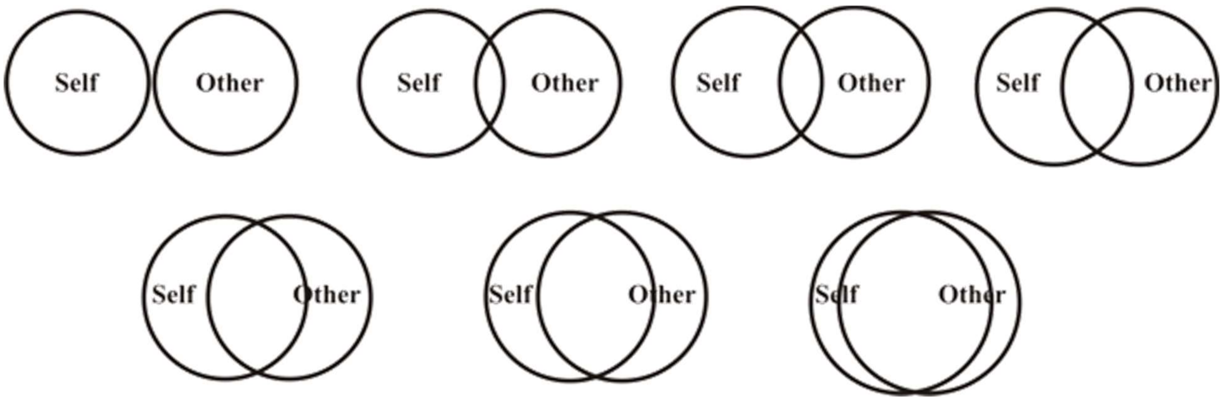

9. Is this the person who caused you to repost #metoo?
    a. Yes
    b. No
10. Do you know anyone who has been falsely accused of sexual violence?
    a. Yes
    b. No
    c. Prefer not to say
11. Think of the person you thought of in the previous question. Which of the below images best represents your relationship with this person?

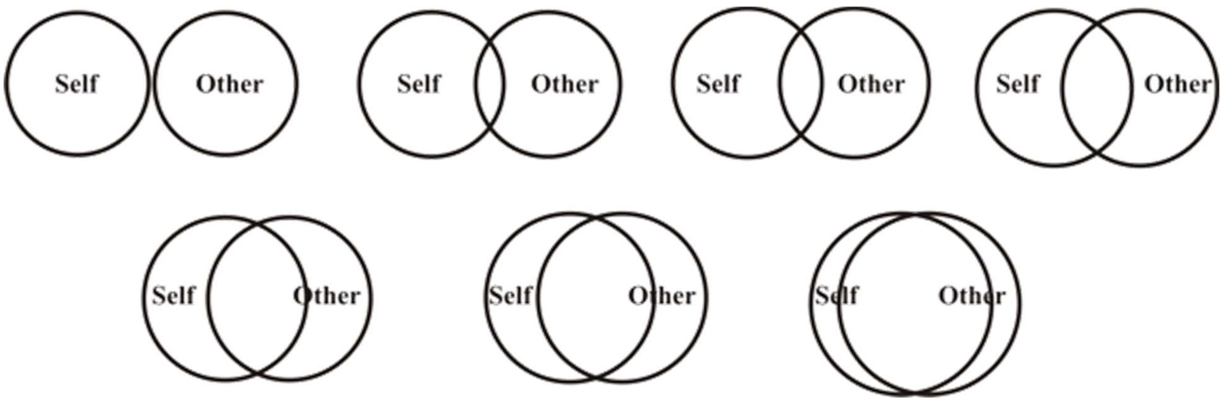

12. Do you know anyone who has falsely accused someone else of sexual violence?
    a. Yes
    b. No
    c. Prefer not to say

13. Think of the person you thought of in the previous question. Which of the below images best represents your relationship with this person?

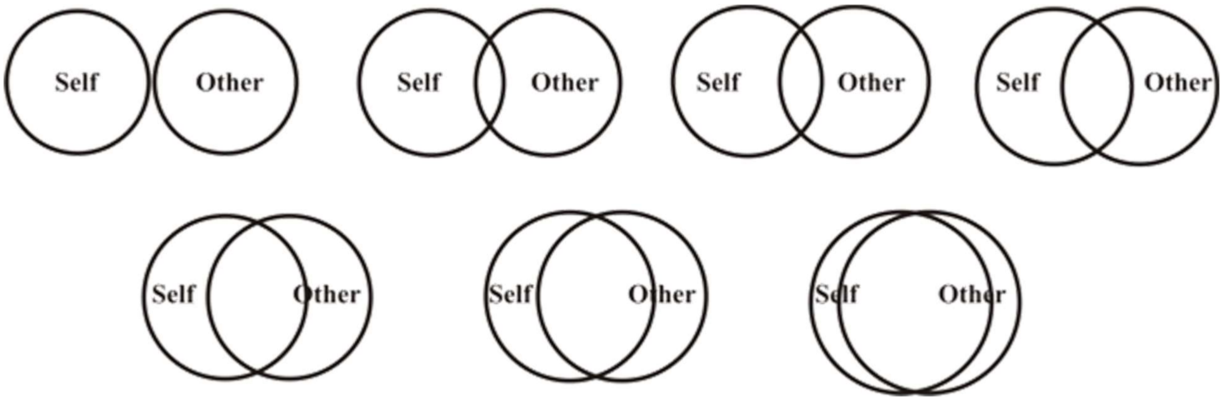

14. Please indicate to what extent you agree whether the #metoo movement is:

Helpful	Strongly Disagree	Somewhat Disagree	Neither Agree nor Disagree	Somewhat Agree	Strongly Agree
Important	Strongly Disagree	Somewhat Disagree	Neither Agree nor Disagree	Somewhat Agree	Strongly Agree
Divisive	Strongly Disagree	Somewhat Disagree	Neither Agree nor Disagree	Somewhat Agree	Strongly Agree
A witch hunt	Strongly Disagree	Somewhat Disagree	Neither Agree nor Disagree	Somewhat Agree	Strongly Agree
Empowering	Strongly Disagree	Somewhat Disagree	Neither Agree nor Disagree	Somewhat Agree	Strongly Agree
Gone too far	Strongly Disagree	Somewhat Disagree	Neither Agree nor Disagree	Somewhat Agree	Strongly Agree

15. Please indicate to what extent the #metoo movement makes you feel:

Supported	Strongly Disagree	Somewhat Disagree	Neither Agree nor Disagree	Somewhat Agree	Strongly Agree
Persecuted	Strongly Disagree	Somewhat Disagree	Neither Agree nor Disagree	Somewhat Agree	Strongly Agree
Worried	Strongly Disagree	Somewhat Disagree	Neither Agree nor Disagree	Somewhat Agree	Strongly Agree

Empowered	Strongly Disagree	Somewhat Disagree	Neither Agree nor Disagree	Somewhat Agree	Strongly Agree
Heard	Strongly Disagree	Somewhat Disagree	Neither Agree nor Disagree	Somewhat Agree	Strongly Agree
Attacked	Strongly Disagree	Somewhat Disagree	Neither Agree nor Disagree	Somewhat Agree	Strongly Agree

CONTROL MANIPULATION

Women 18-24 are more likely to experience sexual violence than any other female demographic in the U.S., according to Rape, Abuse and Incest National Network (RAINN). College students within that age group are three times more likely than the average American woman to be assaulted. Young women within that age group not attending university are four times more likely.

According to a 2014 report from the U.S. Department of Justice, about 80 percent of victims knew their offender. No matter where they occur, these incidences are likely to go unreported, off or on campus. And, 20 percent of both groups cited fear of reprisal as the reason for not reporting.

In the days following allegations against Weinstein, millions took to Twitter, Instagram, Facebook, and other social media to share the hashtag #MeToo. The posts were part of an awareness campaign, aimed at exposing the pervasiveness of sexual harassment and assault, and rape culture in the United States.

"A lot of people have responded to #MeToo by asking why survivors are always the ones who have to come forward and bear our traumas in order for people to see us as human," said Sofie Karasek, 22, to MTV. Karasek organized a candlelight vigil for the survivor advocacy group, End Rape on Campus, in Washington.

"It's also a crucial time to point out that people accused of sexual assault don't just leave college and then disappear into the ether," she said. "They can become powerful people who run companies, like Harvey Weinstein."

"The #MeToo movement highlighted a reality of violence that women, queer, and trans folk already painfully and intimately knew – because we live it day in and day out," Shivani Desai, a national campus organizer for the Feminist Majority Foundation (FMF) said.

"The power and reach of millions of voices provided a national platform, one that emphasized the dangerous culture that affords perpetrators and bigots positions of power and allows them to make harmful decisions."

Adapted from: https://www.voanews.com/a/hashtag-metoo-college-campus-sexual-harassment-assault/4114589.html

FALSE ALLEGATION MANIPULATION

Since the beginning of the #metoo movement, a local police station received 428 reports of sexual assault, compared to only 106 the year before. Of these 428 cases, 427 of the cases went to court and the perpetrator was found guilty. One of the cases turned out to be a false allegation. The man in question, who was falsely accused of rape by his ex-girlfriend has said it will take years to rebuild his life.

David Smith said Lisa Anderson, 23, who was jailed for three years on Monday, left him with nothing when she accused him of rape.

He subsequently lost his job as an IT consultant.

"For me, it is as if someone's house burned down and everything they owned was in it. That is effectively what happened to me," he said.

Ms Anderson told police Mr Smith, 29, had drugged her before attacking her at their home.

"I walked out of the house and I was arrested," he said.

The rape investigation was dropped when officers became suspicious that Ms Anderson had made it up and she was charged, but by this time Mr Smith had lost his job.

Anderson denied charges of perverting the course of justice, theft and fraud but was found guilty by a jury.

Mr Smith said the period after his arrest was difficult mentally. He felt isolated because his former work colleagues knew of the accusations and he was worried about the outcome.

"I think three years is a good sentence for her but for me it is never going to be enough," said Mr Joseph, who now has another job and has moved to a different city.

"It is done - it is over and I am trying to work as hard as I can to get back to where I was.

"It is going to take a number of years, but that is pretty much all I can do."

Adapted from: http://www.bbc.com/news/uk-england-surrey-11676804

Table S1: Demographic Description of Sample

Variable	*N* (%)
Age *M* = 37.74 years, *SD* = 12.92	
**Gender**	
Female	130 (52.0%)
Male	118 (47.2%)
Non-binary	2 (.8%)
**Sexual Orientation**	
Straight/Heterosexual	222 (88.8%)
Bisexual	22 (8.8%)
Gay or Lesbian	4 (1.6%)
Prefer not to say	2 (.8%)
**Education**	
Bachelor Degree	109 (43.6%)
Graduate Degree	63 (25.2%)
Some college but no Degree	39 (15.6%)
Associate Degree	26 (10.4%)
High School Degree or equivalent	13 (5.2%)
**Race**	
White (Non-Hispanic)	167 (66.8%)
White (Hispanic)	31 (12.4%)
Black	27 (10.8%)
Asian	18 (7.2%)
Mixed Race	5 (2.0%)
American Indian or Alaskan Native	2 (.8%)
**Employment**	
Full-time	167 (66.8%)
Part-time	53 (21.2%)
Unemployed	22 (8.8%)
Retired	8 (3.2%)
**Have Children**	
Yes	145 (58.0%)
No	105 (42.0%)

You’re talking with your friend Zoe about #metoo. She was one of the people who reposted the status on her Facebook wall. She tells you she reposted the status because from when she was thirteen until she was fifteen, her mom’s boyfriend would come into her bedroom at night when everyone was asleep. She tells you he would undress and get under the covers with her. He would touch her and rub against her and “do some other stuff”. It only stopped when her mom broke up with him because she had to move to a different city for her job. Zoe has never told anyone about what happened to her, but now that more people are speaking up, she feels that she can too.
